# Evaluation of Survival Outcomes of Endovascular Versus Open Aortic Repair for Abdominal Aortic Aneurysms with a Big Data Approach

**DOI:** 10.3390/e22121349

**Published:** 2020-11-30

**Authors:** Hao Mei, Yaqing Xu, Jiping Wang, Shuangge Ma

**Affiliations:** Department of Biostatistics, Yale University, New Haven, CT 06520, USA; hao.mei@yale.edu (H.M.); yaqing.xu@yale.edu (Y.X.); jiping.wang@yale.edu (J.W.)

**Keywords:** abdominal aortic aneurysm, emulation, deep learning, Medicare data

## Abstract

Abdominal aortic aneurysm (AAA) is a localized enlargement of the abdominal aorta. Once ruptured AAA (rAAA) happens, repairing procedures need to be applied immediately, for which there are two main options: open aortic repair (OAR) and endovascular aortic repair (EVAR). It is of great clinical significance to objectively compare the survival outcomes of OAR versus EVAR using randomized clinical trials; however, this has serious feasibility issues. In this study, with the Medicare data, we conduct an emulation analysis and explicitly “assemble” a clinical trial with rigorously defined inclusion/exclusion criteria. A total of 7826 patients are “recruited”, with 3866 and 3960 in the OAR and EVAR arms, respectively. Mimicking but significantly advancing from the regression-based literature, we adopt a deep learning-based analysis strategy, which consists of a propensity score step, a weighted survival analysis step, and a bootstrap step. The key finding is that for both short- and long-term mortality, EVAR has survival advantages. This study delivers a new big data strategy for addressing critical clinical problems and provides valuable insights into treating rAAA using OAR and EVAR.

## 1. Introduction

Abdominal aortic aneurysm (AAA) is a balloon-like dilatation of the aorta that supplies blood to the body and happens below the chest. Each year, it is estimated that 200,000 people in the U.S. are diagnosed with AAA, and ruptured AAA (rAAA) poses significant clinical and public health challenges [1]. rAAA is associated with an overall mortality rate of over 80%, which causes more than 5000 deaths in the country each year [2,3]. Once rAAA occurs, repairing procedures need to be conducted immediately. In the current clinical practice, there are two main approaches: emergent open aortic repair (OAR) and endovascular aortic repair (EVAR). OAR has a relatively longer history and is still considered as the standard procedure for AAA repair, during which large incisions are unavoidable [4]. EVAR was first successfully conducted and reported in 1994, and only small incisions in the groins are needed [5]. However, this circumvented procedure makes EVAR require more intense monitoring and probable reintervention [6]. Moreover, preoperative imaging and specific anatomic requirements make EVAR less well suitable for emergent rAAA. As suggested in multiple studies [7,8,9,10,11], the preferred minimum invasion but awaited long-term postoperative complications may account for the favorable 30-day mortality but similar or even inferior late survival of EVAR compared to OAR. With the criticalness of rAAA and prevalence of EVAR and OAR, it is of significant interest to objectively evaluate and directly compare their survival outcomes.

In general, to compare the effects of two treatments, the gold-standard approach is to conduct a randomized controlled clinical trial. However, most of the existing clinical trials have focused on patients who have elective/intact AAA (eAAA/iAAA) and excluded those who have rAAA and require emergent care (e.g., OVER [7], DREAM [8]). This is sensible as patients with rAAA cannot bear the prolonged process of eligibility examination, treatment assignment, and finally, surgical procedure, which are non-negligible steps in a clinical trial for bias control but unacceptable for saving lives in a real-world setting.

With the aforementioned concerns, researchers have focused on observational data and analysis to assess the survival outcomes of the two procedures for rAAA patients. Our literature review suggests that quite a few of them have relied on large medical claims databases in particular, including Medicare [6,10,12,13]. In these studies [6,10], regression and other association analysis techniques have been the main tools. It is well recognized that such analyses, even after accounting for confounders, can only lead to conclusions on association, as opposed to the desired cause-and-effect relationship. To overcome such limitations, causal inference techniques [14,15,16] can be adopted. Here we note that, with extensive examinations and comparisons, no approach has been observed to dominate others—it is expected that such an approach may not exist, and different approaches have different pros and cons. In this article, we adopt the emulation approach, which is relatively new but has already been examined in many publications [17,18,19]. With this approach, a clinical trial is explicitly designed and assembled using observational data, and statistical analysis approaches designed for clinical trials can be then adopted, bearing the potential of drawing causal conclusions. Comparatively, the biggest advantage of this approach may be its lucid interpretations.

Built on the emulation strategy, we take a big data analysis approach. Here “big data” is manifested in at least two perspectives. The first is that our effort is built on the Medicare data. The Medicare database is massive, covers the dominating majority of the U.S. senior population, and contains comprehensive information. Compared to for example hospital- and community-based data, Medicare data is advantageous with its unbiased sample selection and relatively uniform and detailed data collection. It has served as the basis of a large number of clinical and public health studies, including those that adopt causal inference analysis techniques [19,20]. More details on the analyzed Medicare data are provided below in Section 2.1. The second big data perspective is that in the analysis of the emulated trial, deep learning techniques are adopted. In “standard” emulation analysis (as well as most if not all analysis of real clinical trials) [18,19], regression (e.g., logistic and Cox) techniques have been adopted. For diverse fields including engineering, business, social science, and others [21,22], the superiority of deep learning techniques in prediction has been well established through a myriad of published studies. Relatively recently, deep learning techniques have been applied to biomedical studies on cancer [23], fracture [24], chronic diseases [25], and cardiovascular diseases [26]. The studied outcomes/phenotypes include continuous [25], categorical [24], and, more recently, survival [27]. It is noted that the existing deep learning analyses of biomedical data are mostly in the association analysis domain.

The overarching goal of this study is to directly compare EVAR versus OAR for rAAA patients and draw conclusions as close to causal as possible, so as to further inform clinical practice. This study may advance from the existing literature in the following aspects:

- It strives to compare the treatment effects of EVAR and OAR under the clinical trial framework, as opposed to the commonly adopted observational data analysis framework. The conclusion so generated may have important and direct clinical implications.
- It advances from the existing emulation analyses by investigating a new disease condition and treatments, which may further expand the paradigm of emulation analysis.
- Deep learning techniques, as opposed to “simple” regressions, are adopted. This study may assist in introducing deep learning to the emulation paradigm, as well as further fostering deep learning research. Specifically, this is the first application of deep learning to the emulation analysis and study of rAAA. Building on the existing deep learning components, we assemble an analysis pipeline that mimics the “propensity score + inverse probability treatment (IPT) weighting Cox regression” approach [18,19].

Looking at a higher level, an “ordinary” clinical trial generates an information set (target), whose most notable characteristic is the balance in information between two treatment arms. In addition, it is usually assumed that such an information set can be sufficiently described using a (semi)parametric model. Information contained in observational data fundamentally differs from the target. As such, a central goal of the emulation approach is to properly carve a subset of information, as large as possible, that mimics the target. With the deep learning analysis approach, the (semi)parametric probabilistic structure can be significantly relaxed. Overall, this study falls into the intersection of information theory and machine learning.

## 2. Methods

This section details the procedures of conducting an emulation study to compare the treatment effects of EVAR and OAR using a big data approach. First, we introduce the Medicare data used in this study in Section 2.1. In Section 2.2 and Section 2.3, we develop protocols of the target randomized clinical trial and the corresponding emulated trial, respectively. Last, we describe the analysis approaches in Section 2.4.

### 2.1. Data Source

As briefly mentioned above, we analyze the Medicare data in this study. Medicare is a federal health insurance program for adults aged 65 years and above, certain younger people with disabilities, and people with end-stage renal disease (permanent kidney failure requiring dialysis or a transplant). As the single largest payer of health care in the U.S., it covers 98% of adults who are over 65 years old, accounts for 99% of death in the elderly population, and generates a huge amount of medical claims data [28]. The Centers for Medicare & Medicaid Services (CMS) offers a wide range of datasets that follow Medicare beneficiaries across multiple care settings. More specifically, it collects over two billion data points per year through reimbursement to hospital care (Medicare Part A), physician and outpatient services (Medicare Part B), drug prescription (Medicare Part D), and other health care claims. It also collects billions of other data points through enrollment information, beneficiary eligibility checks, quality metrics, and calls to 1-800-MEDICARE [29].

For our study, we first retrieve all inpatient claims between 1 January 2011 to 30 September 2015 from the Medicare provider utilization and payment data: hospital care (Part A), which contains detailed information on health services provided in 54 million inpatient episodes for 23 million Medicare beneficiaries. Information contained in each claim includes beneficiary demographics (e.g., age, sex, race), Medicare enrollment status, services provided (up to 25 diagnosis codes and up to 25 procedure codes), and beneficiary death information. More details on such information and how it is utilized in our analysis are provided below.

It is noted that for research purposes, Medicare data can be viewed as publicly available. We only conduct a secondary analysis of the existing deidentified data. As such, no IRB or other approvals are needed.

### 2.2. The Target Randomized Clinical Trial

Under the emulation analysis paradigm [30], one of the first and most important steps is the design of a target randomized clinical trial. For treating rAAA, there is a lack of real clinical trials. As such, similar to in some literature [19], we need to design a hypothetical target trial. The following design has been motivated by relevant observational studies [6,10,11,12,13] and is clinically well grounded.

The target randomized clinical trial aims to compare the short- and long-term all-cause mortality of rAAA patients treated with EVAR and OAR. More specifically, we enroll participants who are diagnosed with rAAA within the enrollment period and exclude those who meet any of the following criteria: (1) the participant is under 65 years old at enrollment; (2) conversion between EVAR and OAR is necessary after randomization; (3) the participant has concurrent conditions of thoracic aneurysms, thoracoabdominal aneurysms, or aortic dissection; and (4) a repair of the thoracic aorta or visceral or renal bypass is considered necessary for the participant. If a participant develops multiple cases of rAAA during the enrollment period, only the first is considered as the primary case and included in analysis. Such criteria have been motivated by observational studies [6,11] and data availability, and have the same level of rigor as a real clinical trial.

The trial enrolls participants from 1 January 2011 to 30 September 2015. After enrollment, each eligible participant is randomized to receive either EVAR or OAR and followed until death, loss to follow-up, or end of the study (30 June 2019). Such decisions have been made with the considerations that both treatments have been extensively adopted in the study period, the enrollment is long enough to ensure a sufficient sample size, and the follow-up is long enough to ensure a sufficient effective sample size.

To assess both short- and long-term mortality after EVAR and OAR, we define two primary outcomes: time from treatment to short-term perioperative mortality and time from treatment to long-term all-cause mortality. The short-term perioperative mortality is defined as death during the index hospitalization or within 30 days of discharge, for which all participants alive at 30 days after discharge are censored. For the long-term all-cause mortality, a subject is censored at loss to follow-up or end of the study (30 June 2019), whichever comes first. The two survival outcomes have different implications and are both critically important [11].

### 2.3. The Emulated Trial

**Figure 1:**
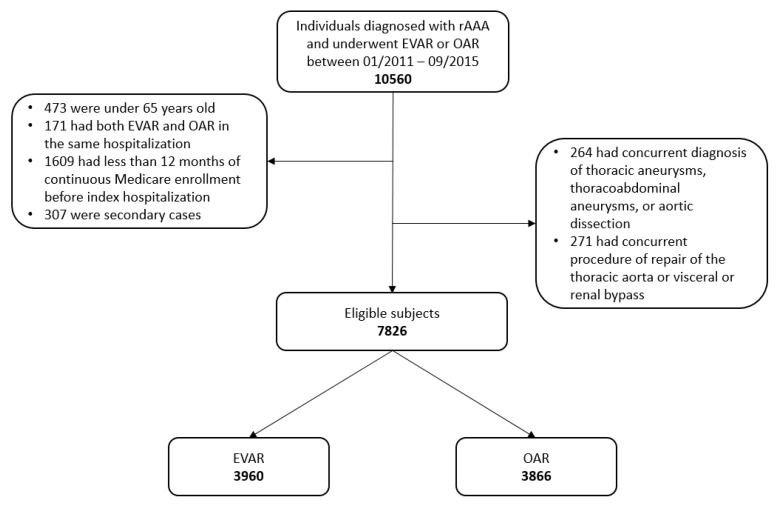
Flowchart of cohort definition.

To emulate the target randomized clinical trial described above, we develop an emulated trial using the Medicare claims data. The strategy closely follows that developed in the emulation literature [19]. First, we identify Medicare beneficiaries who were diagnosed with rAAA and underwent EVAR or OAR between 1 January 2011 and 30 September 2015. We exclude individuals that met any of the following criteria: (1) the individual was under 65 years old at diagnosis; (2) both EVAR and OAR were present in the same index hospitalization, which indicated conversion; (3) concurrent diagnosis codes of thoracic aneurysms, thoracoabdominal aneurysms, or aortic dissection; (4) concurrent procedure codes of repair of the thoracic aorta or visceral or renal bypass; and (5) less than 12 months of Medicare enrollment before the index hospitalization. If a beneficiary had multiple eligible claims, only the first was considered as the primary case and included in analysis. Additional information on patient selection is provided in the flowchart in Figure 1. The relevant International Classification of Diseases, Ninth Revision, Clinical Modification (ICD-9-CM) codes are provided in Appendix A.

We then classify each eligible subject into one of the two treatment groups: EVAR and OAR, based on the procedure he/she actually received. Follow-up information is then extracted for each subject (to death, loss to follow-up, or end of the study which is 30 June 2019). A loss to follow-up is defined as discontinuation of Medicare enrollment. To identify the primary outcomes, we track each study subject from treatment to his/her documented death. We note that there are 5.35% of the study subjects for whom the date of treatment is missing. For these subjects, we use the date of admission to approximate the date of treatment, since rAAA is an emergent condition that needs immediate treatment, and the average lag time between admission and procedure is 0.53 days in our cohort.

### 2.4. Data Analysis

This study has survival outcomes. If this were a real clinical trial, analysis could be conducted using a Cox model. Although balance is expected with proper randomization, to be cautious, in clinical trial analysis, potential confounders are still commonly adjusted. For an emulated trial with a survival outcome, published studies [18,19] suggest the following main analysis steps: (a) conduct a propensity score analysis for treatment using the logistic regression approach, and (b) conduct a Cox regression analysis for survival with IPT weighting. When it can be assumed that all relevant variables are properly included, the first step is a simple parametric regression, and the consistency of parameter estimation can be easily established. The balance in covariate distributions between the two treatment arms for the pseudo sample created by the IPT weighting directly follows. With this balance, the validity of the (weighted) Cox regression follows [31,32].

As briefly mentioned in Section 1, deep learning has demonstrated promising performance with biomedical data. It is of significant interest to apply it to emulation. Equally importantly, the analysis presented in the Supplementary Materials shows that the Cox proportionality assumption is not satisfied. The deep learning approach described below, although has some connections with the Cox model, can be more flexible and less dependent on model assumptions, with its “built-in” flexibility. It consists of the following steps: (a) estimate propensity scores for treatment using a single-layer neural network. This is the counterpart of the logistic regression mentioned above; (b) construct a multi-layer neural network for survival. Advancing from the “standard” deep learning survival, we incorporate weights generated in Step (a), which is the counterpart of the IPT weighted Cox regression mentioned above; and (c) advancing from the existing deep learning literature, we also conduct a bootstrap-type procedure to gain insights into the variation of the neural network weight estimation, which is analogous to the regression coefficient estimation and reveals the treatment effects.

Denote n as the number of independent subjects. For subject i, denote $C_{i}$ as the censoring time and $T_{i}$ as the event time. We observe the right-censored survival outcome $Y_{i}=\min\left(T_{i},\;C_{i}\right)$ and event indicator $d_{i}=I\left(T_{i}\leq C_{i}\right)$ with I(·) being the indicator function. Denote $X_{i}=\left(X_{i1},\;X_{i2},\;\ldots ,\;X_{ip}\right)$ as the baseline covariates and $Z_{i}$ as the binary treatment assignment.

**Step 1**: We employ a single-layer neural network to estimate the propensity score, which is the probability of treatment assignment conditional on the baseline covariates. In particular, the input includes the covariates described in Table 1, with standardization for the continuous variables and coding for the categorical variables. The labels in the data are the binary treatment assignment variables. For the neural network architecture, we use the Rectified Linear Units (ReLU) as the activation function, sigmoid activation function to produce the probability output, and logarithmic loss function (binary cross-entropy). For optimization, a stochastic gradient descent algorithm with Nesterov momentum is used, and a grid search is conducted to tune the learning rate. For such tasks, we adopt the open-source python module keras (https://keras.io). With the outputted propensity score, we compute the IPT weight as its inverse for a subject in one treatment group and the inverse of one minus propensity score for a subject in the other group.

**Step 2:** Here we conduct the IPT weighted survival analysis. The input includes the same set of covariates and treatment indicator as in Step 1, as well as the IPT weights computed above. For subject i, denote $w_{i}$ as the IPT weight and $R_{i}=\left\{j:\;T_{j}>T_{i}\right\}$ as the at-risk set (at time $T_{i}$). We consider a neural network with two hidden layers and the number of nodes determined by tuning. Denote θ as the weights that characterize the network (note that they are not the IPT weights), and $g_{\theta }\left(X_{i},\;Z_{i}\right)$ as the output for subject i. Partly motivated by the loss function under the Cox regression as well as recent deep learning studies, such as DeepSurv, we consider the objective function:

$$l\left(\theta \right)=-\frac{1}{\sum_{i}d_{i}}\sum_{i=1}^{n}d_{i}w_{i}\left[g_{\theta }\left(X_{i},\;Z_{i}\right)-\log\sum_{j\in R_{i}}\exp\left(g_{\theta }\left(X_{j},\;Z_{j}\right)\right)\;\right].$$

For optimization, we adopt a gradient descent approach. ReLU is used as the activation function, and the adaptive moment estimation algorithm (Adam) for gradient descent optimization with a cyclical learning rate method is adopted. We perform a grid search for hyper-parameter tuning. The computational program is developed based on the open-source python module pycox (https://github.com/havakv/pycox).

**Step 3**: A procedure similar to the 0.632 bootstrap for regression analysis [33] is conducted. In particular, 0.632*n* samples are randomly selected from the original data without replacement. With the bootstrapped samples, the above analysis is conducted, and the neural network weight estimates are extracted. This is repeated multiple (e.g., 1000) times to assess the variability of estimates. For regression, the 0.632 bootstrap is equivalent to the “n-out-of-n with replacement” bootstrap. By sampling without replacement, it can reduce ties and computational cost.

In Appendix B, we sketch the algorithm for conducting the Step 3 bootstrap type analysis. The analysis of the whole data amounts to skipping the bootstrapping step and otherwise applying the same procedures.

The above analysis can deliver the following. The first is a propensity score estimate for each subject. If needed, the weights of the neural network can be extracted to help assess the relative contributions of covariates. The second is the survival neural network. For a subject with a set of known confounder values and treatment assignment, it can generate the (relative) survival risk. Most of the existing deep learning studies have treated neural networks as black boxes. As we conduct a clinical trial analysis, the effect of the treatment is of the most essential interest. As such, we retrieve the estimated weights for the treatment indicator and confounders. With the presence of hidden layers, the weight matrices need to be multiplied across layers to obtain the overall contributions. The third product is that, for the (overall) weight of the treatment indicator, the bootstrap type analysis can generate an evaluation of its variability. The same is also applicable to the confounders.

#### Remarks

For a large number of binary data analysis problems, the superiority of neural networks over logistic and other regressions has been established [23,24]. Several recent publications, such as DeepSurv [27] and Cox-nnet [34] and others, seem to suggest similar superiority for survival data. As our goal is to take advantage of the recent deep learning developments, we choose not to “re-establish” the merit of deep learning. We also note that there are multiple “base techniques” for building neural networks. The adopted ones have been shown in recent studies as having a strong mathematical/statistical ground and competitive numerical performance. For example, it has been proved that stochastic gradient descent algorithms can find global minima on the training objective of deep neural networks in polynomial time under mild assumptions [35]. Farrell et al. (2020) established novel nonasymptotic high probability bounds for the fully connected feedforward neural networks with ReLU activations [36]. On the other hand, our literature review suggests that, compared to regression analysis, theoretical research on deep learning remains very rare. Consistency properties (for example, for the weights and bootstrapped estimation) remain unclear. Published literature seems to suggest tremendous challenges. It is beyond our scope to conduct such theoretical investigation.

## 3. Results

### 3.1. Patient Characteristics and Unadjusted Incidences

Our analysis includes 7826 eligible subjects, with 3960 in the EVAR arm and 3866 in the OAR arm. The summary statistics are shown in Table 1. It is observed that the study subjects were slightly younger in the OAR arm and more likely to be white males in both arms. Participants in the OAR arm were healthier with lower percentages of almost all medical conditions (except for two rare conditions: clinically significant lower extremity vascular diseases and renal atherosclerosis). It is also observed that, as time passed by (from year 2011 to 2015), the rAAA patients were more and more likely to receive EVAR. Here we note that, without the IPT weighting, all demographic variables and most medical condition variables are significantly unbalanced between the two treatment arms, highlighting a significant difference between real clinical trials and observational studies. Table 1 also shows the unadjusted incidence rates by treatment. The EVAR arm has a slightly lower unadjusted incidence rate for long-term all-cause mortality and a significantly lower unadjusted incidence rate for short-term perioperative mortality.

### 3.2. Analysis of the Emulated Trial

Prior to analysis, we deleted 15 records with missing measurements (7 in the EVAR arm and 8 in the OAR arm). Analysis was conducted using the approach described in Section 2.4. For the propensity score analysis, the baseline covariates include age, gender, race, year in which repair was performed (this variable has been considered in the published observational studies [6,10]; it is also motivated by the changing rates of EVAR and OAR), and 20 medical conditions, as shown in Table 1 (and with related ICD-9-CM codes in Appendix A). For survival analysis, the same baseline covariates and the treatment indicator are included.

**Figure 2:**
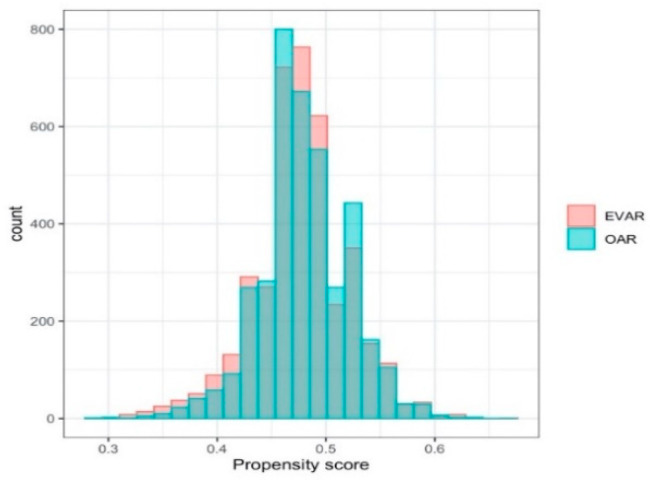
Distribution of propensity score.

For both the propensity score and survival analysis, the obtained fully connected neural network architectures are available from the authors. For the propensity score analysis, the learning rate is tuned as 0.008. The distributions of propensity scores are shown in Figure 2. Minor differences between the two arms are observed.

**Figure 3:**
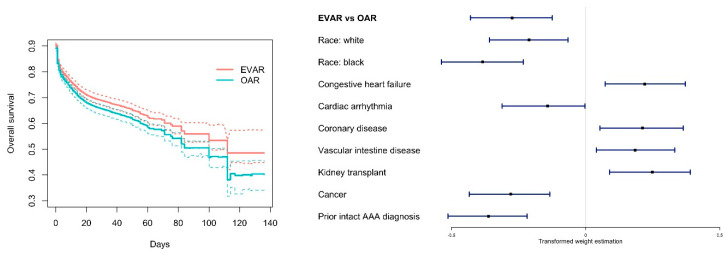
Analysis of short-term mortality. **Left**: estimated survival curves with pointwise 90% confidence intervals. **Right**: forest plot of the estimated weights.

For the analysis of short-term survival, the learning rate for Adam optimizer is tuned as 0.016. The analysis results are summarized in Figure 3. The left panel shows the estimated survival curves, after accounting for IPT weights, for the two treatments separately. With the bootstrap procedure, we are also able to obtain the pointwise 90% confidence intervals. It is noted that this analysis mimics the “familiar” regression analysis and differs from most of the existing deep learning studies, however, it lacks rigorous theoretical justifications that are available for regression analysis. EVAR is observed to have a modest survival advantage, with the lower bounds of its confidence intervals almost coinciding with the upper bounds of OAR’s confidence intervals. Based on the estimated survival curves, we compute the expected survival under EVAR as 83.5 days, compared to 79.2 days under OAR. In the right panel of Figure 3, the forest plot, which shows the medians as well as the 25% and 75% quantile values of the overall estimated weights (“accumulated” over layers), again suggests the survival advantage of EVAR. The right panel of Figure 3 also contains weight information for confounders that demonstrate considerable and “persistent” effects (across the bootstrapped datasets), including race and seven medical conditions.

**Figure 4:**
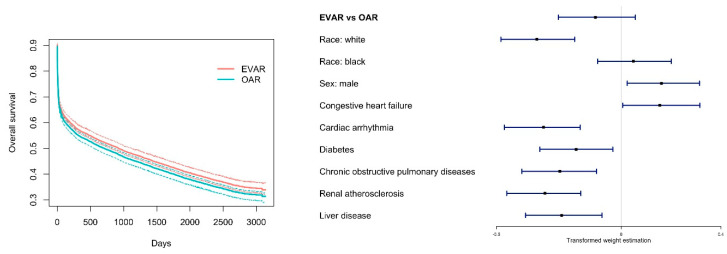
Analysis of long-term mortality. **Left**: estimated survival curves with pointwise 90% confidence intervals. **Right**: forest plot of the estimated weights.

For the analysis of long-term survival, the learning rate for Adam optimizer is tuned as 0.036. The analysis results are summarized in Figure 4, which are parallel to those in Figure 3. The findings are similar to those for short-term survival. Briefly, the left panel suggests some advantages of EVAR, but the pointwise confidence intervals overlap. We compute the expected survival as 1464.2 days under EVAR and 1348.0 days under OAR. The forest plot in the right panel shows that the advantage of EVAR is smaller than that for short-term survival. Confounders that demonstrate considerable and “persistent” effects include race, sex, and six medical conditions.

For comprehensiveness, we also conduct a regression-based analysis. The results are presented in the Supplementary Materials. As the Cox model assumption is violated in both survival analyses, the results cannot be sensibly utilized.

## 4. Discussion

As fully discussed in published literature, the Medicare data has multiple unique advantages. With its broad coverage of the U.S. elderly population, our findings can be applied to this population with high confidence. Although there is no evidence that the relative treatment effects of EVAR and OAR differ by age, sex, and race [11,12,13], application of the findings to the younger U.S. population and populations in other countries/regions should be conducted with cautions. Besides, we have analyzed the Medicare inpatient claims data from 1 January 2011 to 30 June 2019. Both the enrollment and follow-up times are long enough, especially compared to many peer studies [11,12,13].

Recently, deep learning has become increasingly popular in biomedical studies, especially including in the analysis of Medicare and other healthcare data. Beyond those mentioned in the first section, other examples also include Ali et al. (2020), which develops a novel information framework using ensemble deep learning and a feature fusion technique to analyze wearable sensor and electronic medical test data for heart disease prediction [37]. Additional examples include Ali et al. (2020) [38], Jain et al. (2020) [39], and Selden (2020) [40]. The deep learning techniques adopted in this article can fully meet our needs, and it is beyond our scope to comprehensively review/compare existing techniques. We do recognize that it is of interest to explore the applications of other deep learning techniques to the emulation setting.

The emulation strategy has been developed and adopted in quite a few studies. Its pros and cons have been well documented. It is especially noted that, first, emulated trials, although resembling real clinical trials in multiple perspectives, still have notable limitations and cannot replace real clinical trials. Second, there is still a lack of objective comparison and definitive conclusion on its relative performance with respect to other causal inference approaches. Although important, this is beyond the scope of this study. The adopted deep learning methods have been based on certain well-developed components and software programs. Nevertheless, their “combination” and application to the emulation setting and rAAA treatment problem are new and novel. Our analysis has demonstrated how to “replace” regression using deep learning under settings more sophisticated than in the literature. As the “propensity score + survival analysis” strategy and individual components of the deep learning analysis have been more or less developed in the literature, we choose not to methodologically further discuss or conduct more numerical investigations.

Our main finding is that EVAR has advantageous short- and long-term survival. Although the improvement in expected survival is modest, considering the severity of rAAA, it may still have important clinical implications. In the literature, the short-term survival advantage of EVAR has been suggested in multiple observational analyses [6,10,12]. However, there has been a lack of definitive conclusion on the long-term benefit. For example, Behrendt et al. (2017) suggested early survival benefit of EVAR over OAR, which reversed at ~2.5 years of follow-up, for iAAA and rAAA patients in Germany [11]. Schermerborn et al. (2015) observed similar survival of the two procedures after 3 years from initial surgery for iAAA patients in the Medicare population [6]. And a 15-year follow-up resulted from the EVAR-1 trial indicated that EVAR had inferior late survival compared to OAR [9]. Multiple factors can contribute to the differences observed in the aforementioned and other studies. First, the studied populations have different characteristics. Second, the analysis strategies also differ, with our strategy closer to a controlled clinical trial. It is also noted that the study periods are different. Although there is still no indication of temporal variation in treatment effects, related confounders may change over time.

Besides treatment, our analysis also suggests that race, gender, and certain medical conditions are associated with survival after EVAR and OAR among rAAA patients. While most observational studies that compare EVAR and OAR match study subjects or adjust for potential confounders, there is a lack of attention on how these variables may impact survival after rAAA. We have found that compared to other races, the white race is associated with lower short- and long-term mortality, and the black race is associated with lower short-term mortality. This race difference has been insufficiently studied in the literature. It can be caused by genetic effects (considering that genetic factors contribute to many cardiovascular diseases), lifestyle, cultural factors, access to care, and other factors that may confound survival. While Egorova et al. (2011) observed significantly worse outcomes after EVAR and OAR for female patients, we have found no gender difference in short-term mortality and male associated with higher long-term mortality [12]. One contributing factor is the difference in analysis technique: Egorova et al. (2011) compared the observed survival with expected survival in a life table [12], while we have conducted a more comprehensive adjusted analysis. Lastly, we have identified certain medical conditions as associated with survival. What may seem counterintuitive is that some medical conditions are found as negatively associated with mortality. For example, it is found that prior intact AAA diagnosis decreases short-term mortality risk after rAAA, and the presence of cardiac arrhythmia increases both short- and long-term survival. One plausible explanation is that patients with related medical conditions are more likely to have regular hospital visits and more access to healthcare services, which may lead to more timely detection of emergent rAAA. For example, it is noted in Edwards et al. (2014) that patients who had a prior diagnosis of intact AAA were less commonly admitted through the emergency department, and were more commonly transferred between hospitals before treatment, which was associated with better survival [10]. Dardic et al. (1998) also found that the presence of hypertension, diabetes, and COPD was correlated with a statistically significant lower mortality rate, whereas the presence of smoking, heart disease, and renal disease was correlated with a statistically insignificant lower mortality rate after the diagnosis of rAAA [41].

This study inevitably has limitations. In particular, there is limited information covered by the Medicare data [28,29]. Therefore, the treatment arms may be imbalanced on unmeasured confounders such as over-the-counter drug uses and patients’ socioeconomic information. We note that this limitation is shared by other emulation studies and analysis of observational data. Moreover, due to limited data access, this study examines rAAA patients’ inpatient treatments from 1 January 2011 to 30 September 2015. With the special nature of rAAA, inpatient claims should be able to catch the dominating majority of the cases. However, it remains unclear whether utilization of other clinical settings (e.g., emergency room or outpatient) affects the treatment effects of EVAR and OAR. Also, although there is no indication that the treatment effects have temporal variations, it may still be of interest to examine more extensive data. In addition to data limitations, it is well documented in the literature that the emulation approach, while being lucidly interpretable, has limitations [14,18,19]. For example, the approach can only emulate target trials without blind assignment. Given the specific natures of EVAR and OAR, lack of blinding is not necessarily a limitation for this study. However, future studies using the emulation approach should be cautious of these limitations.

With the possibility of more extensive data, it is of future interest to investigate the aforementioned potential confounders that are unmeasured in this study, the effects of other clinical settings on the treatment effects of EVAR and OAR after rAAA, and the potential temporal variations. Moreover, it is also postponed to future work to conduct a direct comparison of the emulation approach and other causal inference approaches using the large-scale Medicare data.

## 5. Conclusions

This study has suggested certain short- and long-term survival advantage of EVAR over OAR for rAAA patients. It has also further advanced the emulation and deep learning techniques for analyzing data mined from large medical record databases. Both the medical findings and analytic developments can complement the existing literature and be of interest to stakeholders at multiple levels.

## Figures and Tables

**Table 1:** Descriptive characteristics of the study cohort.

	EVAR (N = 3930)	OAR (N = 3866)	*p*-Value *
**Demographic**			
Age, mean(sd)	78.03 (7.52)	76.59 (6.90)	<0.0001
Male	3023 (76.34)	2786 (72.06)	<0.0001
Race			0.0030
White	3588 (90.77)	3550 (92.02)	
Black	249 (6.30)	178 (4.61)	
Other	116 (2.93)	130 (3.37)	
**Medical conditions**			
Congestive heart failure	464 (11.72)	299 (7.73)	<0.0001
Cardiac arrhythmia	596 (15.05)	438 (11.33)	<0.0001
Valvular disease	199 (5.03)	172 (4.45)	0.2304
Coronary disease	758 (19.14)	603 (15.60)	<0.0001
Diabetes	329 (8.31)	256 (6.62)	0.0045
Hypertension	1250 (31.25)	1078 (27.88)	0.0004
Chronic obstructive pulmonary diseases	707 (17.85)	584 (15.11)	0.0011
Clinically significant lower extremity vascular diseases	26 (0.66)	27 (0.70)	0.8215
Renal atherosclerosis	20 (0.51)	27 (0.70)	0.2684
Vascular intestine disease	7 (0.18)	2 (0.05)	0.1027
Renal failure	493 (12.45)	358 (9.26)	<0.0001
Other renal diseases	3 (0.08)	1 (0.03)	0.3289
Kidney transplant	4 (0.10)	3 (0.08)	0.7291
Liver disease	33 (0.83)	30 (0.78)	0.7766
Cerebrovascular diseases and paralysis	93 (2.35)	67 (1.73)	0.0544
Other neurological diseases	153 (3.86)	114 (2.95)	0.0258
Hyperlipidemia	817 (20.63)	687 (17.77)	0.0013
Cancer	132 (3.33)	87 (2.25)	0.0037
Rheumatoid arthritis	76 (1.92)	39 (1.01)	0.0008
Prior intact AAA diagnosis	511 (12.90)	440 (11.38)	0.0393
**Other**			
Year in which repair was performed			<0.0001
2011	808 (20.40)	1013 (26.20)	
2012	869 (21.94)	913 (23.62)	
2013	819 (20.68)	785 (20.31)	
2014	837 (21.14)	701 (18.13)	
2015	627 (15.83)	454 (11.74)	
**Outcome (followed until death, loss to follow-up, or 06/30/2019)**			
All-cause mortality	2430 (61.36)	2542 (65.75)	<0.0001
Perioperative mortality (in-hospital or 30 days after discharge)	1107 (27.95)	1704 (44.08)	<0.0001

* p-values based on *t*-tests for continuous variables and Chi-squared test for categorical variables.

## Supplementary Materials

The following are available online at https://www.mdpi.com/1099-4300/22/12/1349/s1.

## Appendix A

**Table A1:** International Classification of Disease, 9th edition, Clinical Modification (ICD-9-CM) codes for identifying eligible individuals and defining confounders (one year of medical history).

**Variable**	**ICD-9-CM Code ***
**Inclusion**	
Ruptured abdominal aortic aneurysm	441.3
Endovascular aortic repair	39.71
Open aortic repair	38.44, 39.25, 39.52, 38.34, 38.64, 38.40, 38.60
**Exclusion**	
Thoracic aneurysms	441.1, 441.2
Thoracoabdominal aneurysms	441.6, 441.7
Aortic dissection	441.00-441.03
Repair of the thoracic aorta	38.35, 38.45, 39.73
Visceral or renal bypass	38.46, 39.24, 39.26
**Medical history**	
Congestive heart failure	398.91, 402.01, 402.11, 402.91, 404.01, 404.03, 404.11, 404.91, 404.13, 404.93, 425.4, 425.5, 425.7, 425.8, 425.9, 428.0, 428.1, 428.20, 428.22, 428.30, 428.32, 428.40, 428.42, 428.9
Cardiac arrhythmia	426.0, 426.10, 426.11, 426.12, 426.13, 426.7, 426.9, 427.0, 427.1, 427.2, 427.3, 427.9, V45.0, V53.3
Valvular disease	093.2, 394, 395, 396, 397, 424, V42.2, V43.3
Coronary disease	412, 413, 414, 429.2
Diabetes	250
Hypertension	401, 402, 403, 404, 405
Chronic Obstructive Pulmonary diseases	416, 417.9, 490, 491, 492, 493, 494, 495.0, 495.1, 495.2, 495.3, 495.4, 495.5, 495.6, 495.8, 495.9, 496, 500, 501, 502, 503, 504, 505, 506.0, 506.2, 506.4, 506.9, 508.1, 508.8, 508.9
Clinically significant lower extremity vascular diseases	440.22, 440.23, 440.24, 440.3, 444.22, V43.4,
Renal atherosclerosis	440.1
Vascular intestine disease	557.1
Renal failure w dialysis	V45.1, V56.0, V56.1, V56.2, V56.3, V56.8, 585.6, 39.95 (w/o 586)
Renal failure without dialysis	403.01, 403.11, 403.91, 404.02, 404.03, 404.12, 404.13, 404.92, 404.93, 585 (w/o 585.6), 588.0
Other renal diseases	582, 583.0, 583.1, 583.2, 583.4
Kidney transplant	V420
Liver disease	070.22, 070.23, 070.32, 070.33, 070.44, 070.54, 070.9, 456.0, 456.1, 571, 572.1, 572.2, 572.3, 572.4, 572.8, 573.0, 573.1, 573.8, 573.9
Cerebrovascular diseases and paralysis	342, 344.1, 344.3, 344.4, 344.5, 344.9, 437.0, 438
Other neurological diseases	330, 331, 332, 333, 334.0, 334.1, 334.2, 334.4, 334.8, 335.0, 335.1, 335.2, 335.8, 335.9, 336.0, 336.2, 343, 344.0, 348.1, 348.3, 344.2, 344.6, 345, 437.3, 437.4, 437.5, 437.6, 437.7
Hyperlipidemia	272
Cancer	140, 141, 142, 143, 144,145, 146, 147, 148, 149, 150, 151, 152, 153, 154, 155, 156, 157, 158,159, 160, 161, 162, 163, 164, 165, 170, 171,172, 174, 175, 176, 179, 180, 181, 182, 183, 184, 185, 186, 187, 188, 189, 190, 191, 192, 193, 194, 195, 196, 197, 198, 199, 200, 201, 202, 203.0, 238.6
Rheumatoid arthritis	446, 701.0, 710.0, 710.1, 710.2, 710.3, 710.4, 710.8, 710.9, 711.2, 719.3, 714,720, 725, 728.5, 728.89
Prior intact AAA diagnosis	441.4, without mention 441.3

* Primary or any secondary diagnosis/procedure code.

## Appendix B

**Algorithm A1.** Algorithm for the bootstrap type analysis (Step 3).
1. Initialize b=0.
2. Update b=b+1, randomly sample 0.632*n* subjects without replacement from the original data and conduct the following procedure.
**Step 1.** Estimate the propensity score.
• Construct a simple multilayer perceptron with one hidden layer, ReLU and sigmoid activations, and binary cross-entropy as the loss function.
• For a fixed learning rate from a vector of hyper-parameter values: ○ Compile the model using a stochastic gradient descent optimizer with Nesterov momentum. ○ Compute the classification accuracy as the metrics for model selection.
• Select the best model with the highest accuracy from the above grid search, and calculate the IPT weight by transforming the estimated propensity score.
**Step 2.** Assess the treatment effects using the IPT weighted survival analysis.
• Construct a multilayer perceptron with two hidden layers, ReLU activations, and the proposed loss function l(θ).
• For a fixed learning rate, the number of nodes in hidden layers from a grid of hyper-parameter values: ○ Compile the model using Adam optimizer. ○ Estimate the weights θ, and compute the concordance statistic for model selection.
• Select the best model with the largest concordance, and obtain the effects of treatment and other confounders by multiplying the estimated weights across layers.
3. Repeat until b=B is large enough (e.g., B=1000).
