# Supplementary material for: Evaluation of Survival Outcomes of Endovascular Versus Open Aortic Repair for Abdominal Aortic Aneurysms with a Big Data Approach
Source: Entropy (Basel). 2020 Nov 30;22(12):1349. doi: 10.3390/e22121349 (PMC7759828; doi:10.3390/e22121349)
Supplement: Supplementary file 1 [file entropy-22-01349-s001.pdf]

Supplementary Materials

In "standard" clinical trial analyses as well as emulated trial analyses [1,2], regression techniques have generally been adopted. Here to complement the analysis presented in the main text, we also conduct regression-based analysis. The overall strategy is similar to that presented in Section 2.4. The first step is to estimate the propensity score using a logistic regression. The same set of variables as presented in Table 1 is included. Then we conduct the IPT weighted Cox regression analysis. Both steps can be realized using existing R functions. For inference, we directly use the P values generated by Cox regression. There is hence no need for bootstrap.

The estimated propensity scores are shown in Figure S1.

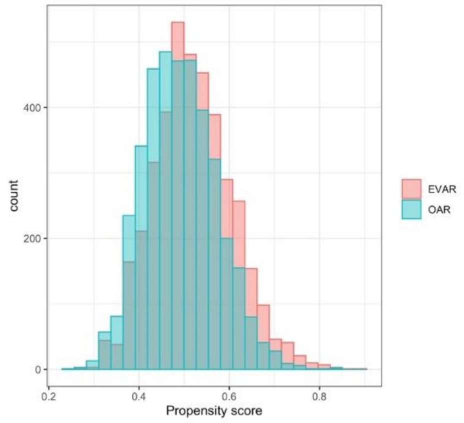

Figure S1. Distribution of propensity score using logistic regression.

In the analysis of short-term survival, the estimated coefficient of EVAR is -0.611, with a standard error of 0.027 and a p-value less than 0.001. Other significant variables include age, hypertension, chronic obstructive pulmonary diseases, and prior intact AAA diagnosis. More detailed estimation and inference results are available from the authors. We test the proportional hazards assumption, and the global Chi-squared test returns a p-value less than 0.001, *suggesting a violation of model assumptions*. We further plot the scaled Schoenfeld residuals for treatment in the left panel of Figure S2 and observe that the residuals are correlated with time. The plot of deviance in the right panel of Figure S2 also suggests a violation of model assumptions.

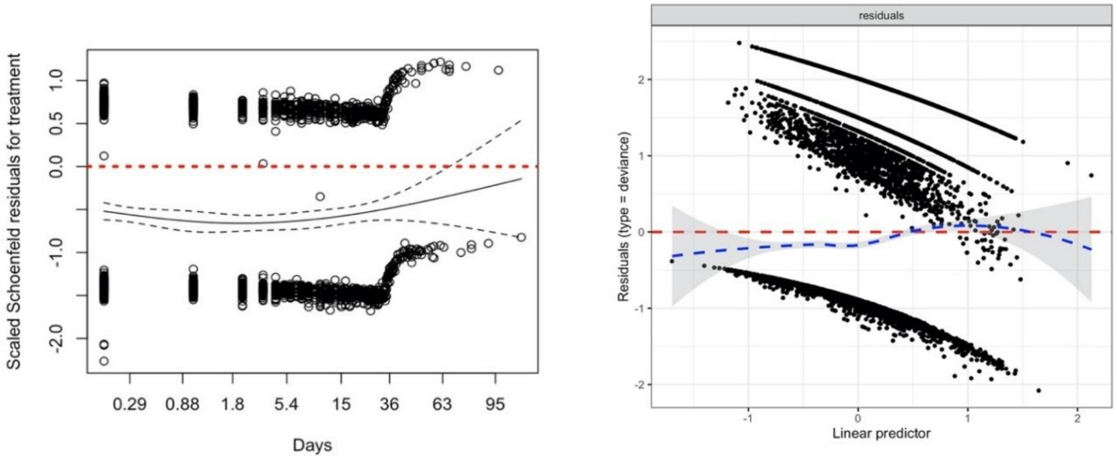

(**a**) (**b**)

Figure S2. Analysis of short-term survival using Cox regression. Left: Scaled Schoenfeld residuals for treatment. Right: Deviance residuals.

In the analysis of long-term survival, the estimated coefficient of EVAR is -0.317, with a standard error of 0.002 and a p-value less than 0.001. Other significant variables include age, hypertension, chronic obstructive pulmonary diseases, and prior intact AAA diagnosis. The global Chi-squared test returns a p-value less than 0.001, which, along with Figure S3, suggests that the Cox model assumptions are not satisfied.

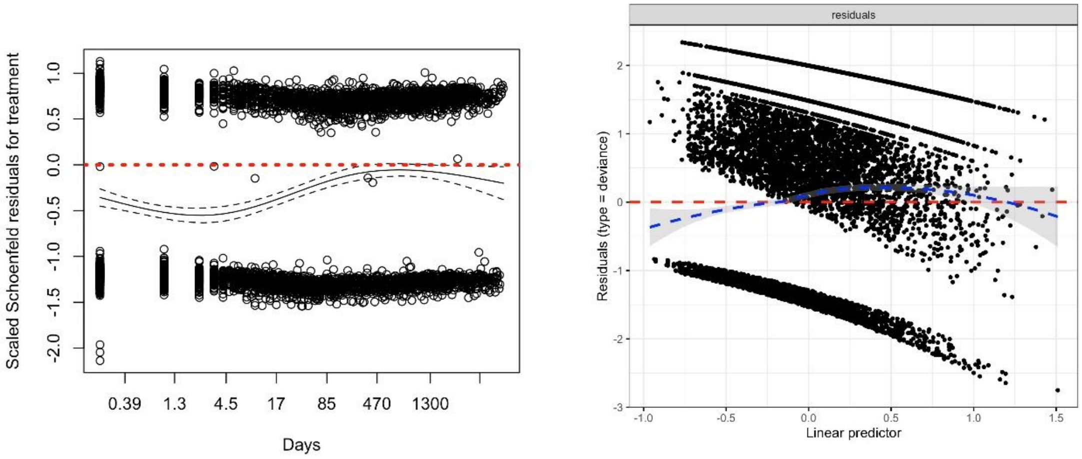

(**a**) (**b**)

Figure S3. Analysis of long-term survival using Cox regression. Left: Scaled Schoenfeld residuals for treatment. Right: Deviance residuals.

Overall, we present the regression-based results for completeness but note that, with the violations of model assumptions, their results should not be utilized.

Reference:

1. Schermerhorn, M.L.; Cotterill, P. Endovascular vs. Open Repair of Abdominal Aortic Aneurysms in the Medicare Population. *New England Journal of Medicine* **2008**, *358*, 464–474.
2. Edwards, S.T.; Marc L. Schermerhorn; A. James O'Malley; Rodney P. Bensley; Rob Hurks; Philip Cotterill; Bruce E. Landon Comparative effectiveness of endovascular versus open repair of ruptured abdominal aortic aneurysm in the Medicare population. *Journal of Vascular Surgery* **2014**, *59*, 575–582.
